# Open-loop analysis on sympathetically mediated arterial pressure and urine output responses in spontaneously hypertensive rats: effect of renal denervation

**DOI:** 10.1186/s12576-021-00798-x

**Published:** 2021-04-20

**Authors:** Toru Kawada, Takuya Nishikawa, Satoru Suehara, Satoshi Sawada, Tetsuo Tanaka, Minako Uenohara, Hiromi Yamamoto, Masaru Sugimachi

**Affiliations:** 1 Department of Cardiovascular Dynamics, National Cerebral and Cardiovascular Center, Osaka, 564-8565 Japan; 2 Corporate R&D Center, Terumo Corporation, Kanagawa, 259-0151 Japan; 3 Department of Cardiology, Kurashiki Central Hospital, Ohara HealthCare Foundation, Okayama, 710-8602 Japan

**Keywords:** Pressure diuresis, Sympathetic nerve activity, Arterial pressure, Open-loop analysis, Equilibrium diagram

## Abstract

Primary acute sympathetic activation (PASA) causes a subsequent arterial pressure (AP) elevation. In this case, an antidiuretic effect via the renal innervation and pressure diuresis can act antagonistically on the kidneys. We examined the effect of PASA on urine output in spontaneously hypertensive rats (SHR) 4–7 days after unilateral renal denervation (RDN) (*n* = 9). The slope of the plot of urine flow versus AP was positive (0.120 ± 0.031 μL min^−1^ kg^−1^ mmHg^−1^) on the intact side, but it was less than 1/3 of the slope observed previously in normotensive Wistar–Kyoto rats (WKY). RDN did not normalize the slope of urine flow versus AP (0.179 ± 0.025 μL min^−1^ kg^−1^ mmHg^−1^, *P* = 0.098 versus the intact side). The urine flow at the operating point of the AP tended to be greater on the denervated than the intact side (29.0 ± 1.8 vs. 25.3 ± 1.9 μL min^−1^ kg^−1^, *P* = 0.055). The percent increase (17.2 ± 7.2%) was not different from that observed previously in WKY. Although high-resting sympathetic nerve activity is prerequisite for maintaining hypertension in SHR, the effect of sympathetic innervation on the urine output function was not greater than that in WKY.

## Background

Urine output control is essential for the long-term regulation of arterial pressure (AP). A decrease in urine output promotes body fluid retention and contributes to the increase of AP, and vice versa. Renal sympathetic activation exerts an antidiuretic effect via the release of renin, renal vasoconstriction, and sodium and water reabsorption [1]. It has been shown that renal sympathetic activation reduces urine output under a constant renal perfusion pressure in anesthetized dogs [2]. However, the relationship between sympathetic nerve activity (SNA) and urine output may not always be negative. During primary acute sympathetic activation (PASA) that induces a subsequent AP elevation, an increase in renal perfusion pressure can promote diuresis and antagonize the antidiuretic effect via renal sympathetic innervation.

In a previous study, we demonstrated that the urine output positively correlated with SNA during PASA in normotensive Wistar–Kyoto rats (WKY) [3]. It was concluded that the pressure diuresis mechanism outweighs the antidiuretic effect mediated by the renal sympathetic nerve during PASA. In that study, unilateral renal denervation (RDN) caused a higher urine output at the operating point of AP in the denervated than in the intact kidney. Sympathetic activation is one of the key factors of the pathogenesis of hypertension. We hypothesized that the contribution of the renal sympathetic control to the urine output function would be greater in spontaneously hypertensive rats (SHR). If so, the urine output increase by RDN would be augmented in SHR. To test the hypothesis, we examined the urine output change during PASA induced by baroreceptor pressure inputs in anesthetized SHR. The experiment was performed on SHR 4–7 days after unilateral RDN, and the urine output was compared between the intact and denervated sides.

## Methods

### Ethical approval

Male SHR were purchased from Japan SLC at 12 weeks of age. The rats were cared for in strict accordance with the Guiding Principles for the Care and Use of Animals in the Field of Physiological Sciences, which has been approved by the Physiological Society of Japan. The Animal Subjects Committee at the National Cerebral and Cardiovascular Center reviewed and approved all experimental protocols.

### Renal denervation

Unilateral RDN was performed on nine rats under isoflurane anesthesia using a sterile preparation. Through a flank incision, visible renal nerves were sectioned under a dissecting microscope, and a solution of 10% phenol in ethanol was applied around the renal vessels [4]. The incision was closed, and butorphanol tartrate was injected intramuscularly for postoperative analgesia. Six rats underwent a right RDN procedure, and remaining three rats underwent a left RDN procedure. If the observed difference in the urine output function was attributed solely to the laterality, the inclusion of data from the denervation on the different side would have canceled the statistical difference. After the unilateral RDN, each rat was housed individually and given free access to standard laboratory chow and water.

At the end of the acute experiment described below, tissue samples obtained from the kidneys (100–200 mg) were frozen at − 80 °C. Later, the tissue norepinephrine concentration was measured with a high-performance liquid chromatography system (Eicom, Japan). We defined the RDN as successful when norepinephrine was depleted to less than 10% in the denervated side relative to the intact side. All nine rats met this criterion (0.3–9.0%).

### Preparation of acute experiment

Four-to-seven days after the RDN, the rats (295–338 g) were anesthetized by intraperitoneal injection (2 mL/kg) of a mixture of urethane (250 mg/mL) and α-chloralose (40 mg/mL). For the maintenance of anesthesia, an 18-fold diluted solution of the anesthetic mixture was administered (2 mL kg^−1^ h^−1^) via the right femoral vein. Ringer's lactate solution was also infused (4 mL kg^−1^ h^−1^) via the left femoral vein for fluid maintenance. Systemic AP was measured via a polyethylene catheter (PE50, Becton Dickinson and Company, MD, USA) that was inserted into the right femoral artery. Heart rate (HR) was detected from the AP waveform. The rats were mechanically ventilated with oxygen-enriched room air. The body temperature of the rat was maintained at approximately 38 °C with a heating pad and a lamp.

As a proxy of systemic SNA, the splanchnic sympathetic nerve was selected, because the control of splanchnic vascular bed plays an essential role in systemic AP regulation [5]. A pair of stainless-steel wire electrodes (AS633, Cooner Wire, CA, USA) were attached to a postganglionic branch of the left splanchnic sympathetic nerve and fixed with silicone glue (Kwik-Sil, World Precision Instruments, FL, USA). The SNA was quantified via full-wave rectification and low-pass filtering with a cut-off frequency of 30 Hz. At the end of the experiment, a ganglionic blocker hexamethonium bromide (60 mg/kg) was injected intravenously to assess the noise level of the SNA recording.

Before isolating the carotid sinus baroreceptor regions, we measured AP and HR for more than 5 min. Thereafter, bilateral carotid sinus baroreceptor regions were isolated from the systemic circulation [6, 7], and carotid sinus pressure (CSP) was controlled with a servo-controlled piston-pump system (ET-126, Labworks, Costa Mesa, CA, USA). The aortic depressor nerves and vagal nerves were sectioned bilaterally to prevent reflexes other than the carotid sinus baroreflex from confounding the results.

A polyethylene tube (KN-392-SP 8, inner diameter: 0.2 mm, outer diameter: 0.5 mm, Natsume, Japan) was inserted into each ureter via a horizontal abdominal incision. Urine from each ureter was collected in a 1-mL syringe placed vertically on the lateral side of a surgical table. The top of the syringe was positioned just below the surface of the table [3]. The urine volume was calculated based on the hydrostatic pressure of the collected urine relative to that of 1-mL physiological saline.

### Protocol of acute experiment

The CSP was first decreased to 60 mmHg and increased stepwise up to 220 mmHg in 20 mmHg increments. The CSP step duration was either 60 or 90 s, which was longer than the total response time of the urine flow (UF) change during a short-term AP perturbation in rats (17.6–26.7 s) [8]. The CSP step input was repeated two-to-three times.

The fluid maintenance started after the venous catheterization, and the entire surgical preparation lasted approximately 90 min. After the completion of the surgical preparation, another 30 min was allowed before the CSP step input protocol was initiated. Hence, the differences in the hydration status among rats might have reduced, if not completely disappeared.

### Blood, urine, and renal tissue samples

A blood sample was extracted at the end of the acute experiment. After centrifugation, the plasma was frozen at − 80 °C. Cumulated urine from each kidney was also frozen at − 80 °C. The sodium and creatinine concentrations of the plasma and urine samples were measured (Hachioji Laboratories, SRL Inc., Japan).

### Data analysis

Data were stored at 1000 Hz on a laboratory computer system via a 16-bit analog-to-digital converter. The mean SNA and AP values at each CSP level were calculated during the last 10 s of each step. The mean values at the same CSP level from the multiple CSP step inputs were then averaged. The SNA was normalized with the value at CSP of 60 mmHg (100%) and the value after the ganglionic blockade (0%). The UF (in μL/min) at each step was derived from the increment of the urine volume from the preceding step. When the step duration was 90 s, the increment was divided by 1.5. The normalized urine flow (nUF, in μL min^−1^ kg^−1^) was defined as the UF value divided by the body weight of the rat.

The total reflex arc (the AP change as a function of CSP) and the neural arc (the SNA change as a function of CSP) were quantified by fitting a four-parameter logistic function to the data [9]:

1 $$y=\frac{{P}_{1}}{1+\mathrm{exp}\left[{P}_{2}\left(CSP-{P}_{3}\right)\right]}+{P}_{4},$$

where *P*_1_ is the response range, *P*_2_ is the slope coefficient, *P*_3_ is the midpoint pressure on the CSP axis, and *P*_4_ is the lower plateau of the sigmoid curve.

The peripheral arc (the AP change as a function of SNA) was quantified by linear regression. The operating-point SNA and AP values were estimated from the intersection point between the fitted neural and peripheral arcs on a baroreflex equilibrium diagram [10–12].

The SNA–nUF relationship and the AP–nUF relationship were also quantified by linear regression. The nUF at the operating-point AP, which was estimated on the regression line of the AP–nUF relationship, was used to calculate creatinine clearance in each kidney. Fractional sodium excretion was calculated from the creatinine and sodium concentrations in the plasma and cumulated urine.

### Statistical analysis

The data are expressed as mean ± SE values. Differences between the intact and denervated sides were examined using the Wilcoxon signed-rank test [13]. In the discussion section, we also performed statistical comparisons of the data between SHR in the present study (*n* = 9) and WKY in our previous study (*n* = 10) using the Mann–Whitney test. The difference was considered significant at *P* < 0.05.

## Results

**Fig. 1:**
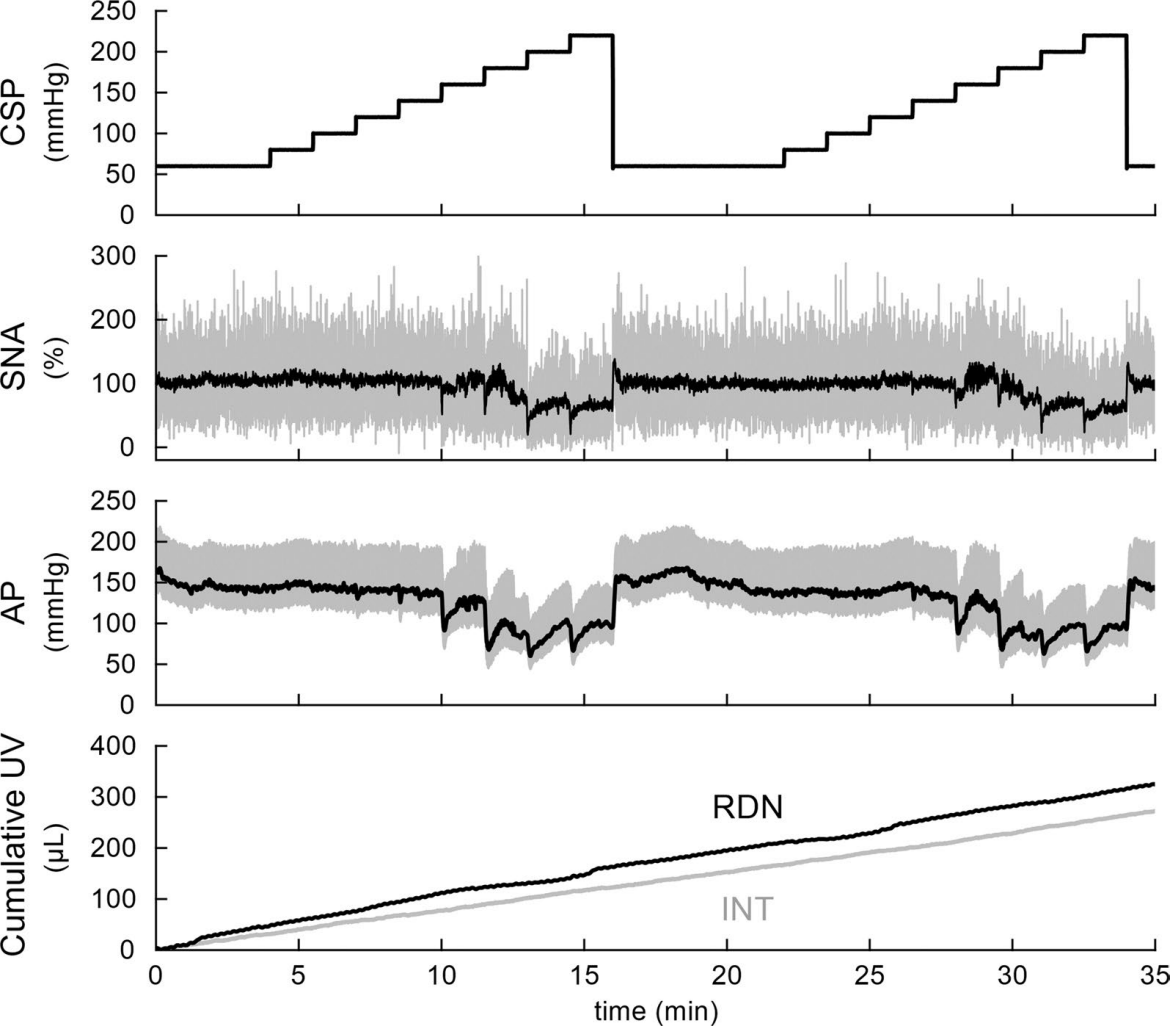
An example time-series obtained from one rat. The carotid sinus pressure (CSP) was changed in a stepwise manner with a step duration of 90 s. Sympathetic nerve activity (SNA) and arterial pressure (AP) decreased in response to CSP when the CSP was above 140 mmHg. Gray and black lines in the SNA plot indicate 10-Hz resampled and 2-s moving average signals, respectively. Gray and black lines in the AP plot indicate 200-Hz resampled and 2-s moving average signals, respectively. Urine volume (UV) is displayed as a 10-Hz resampled signal. The UV was greater on the renal denervation (RDN) side compared with the intact (INT) side, and the difference increased as time elapsed

Typical time-series obtained in one rat are shown in Fig. 1. Increasing CSP up to 140 mmHg did not affect SNA or AP. Increasing CSP above 140 mmHg caused SNA suppressions, but the mean SNA remained at approximately 50% in response to the CSP of 220 mmHg. The AP reductions were observed in response to the CSP steps above 140 mmHg, but the response to each step was not maintained during the 90 s step duration. The urine volume in the intact side increased nearly linearly with time. The urine volume was greater on the denervated than the intact side, and the difference increased as time elapsed. The urine volume in the denervated side showed low-frequency fluctuation that did not seem to be related to AP changes. This fluctuation was not a consistent observation across rats and disappeared during the analysis of pooled data.

**Fig. 2:**
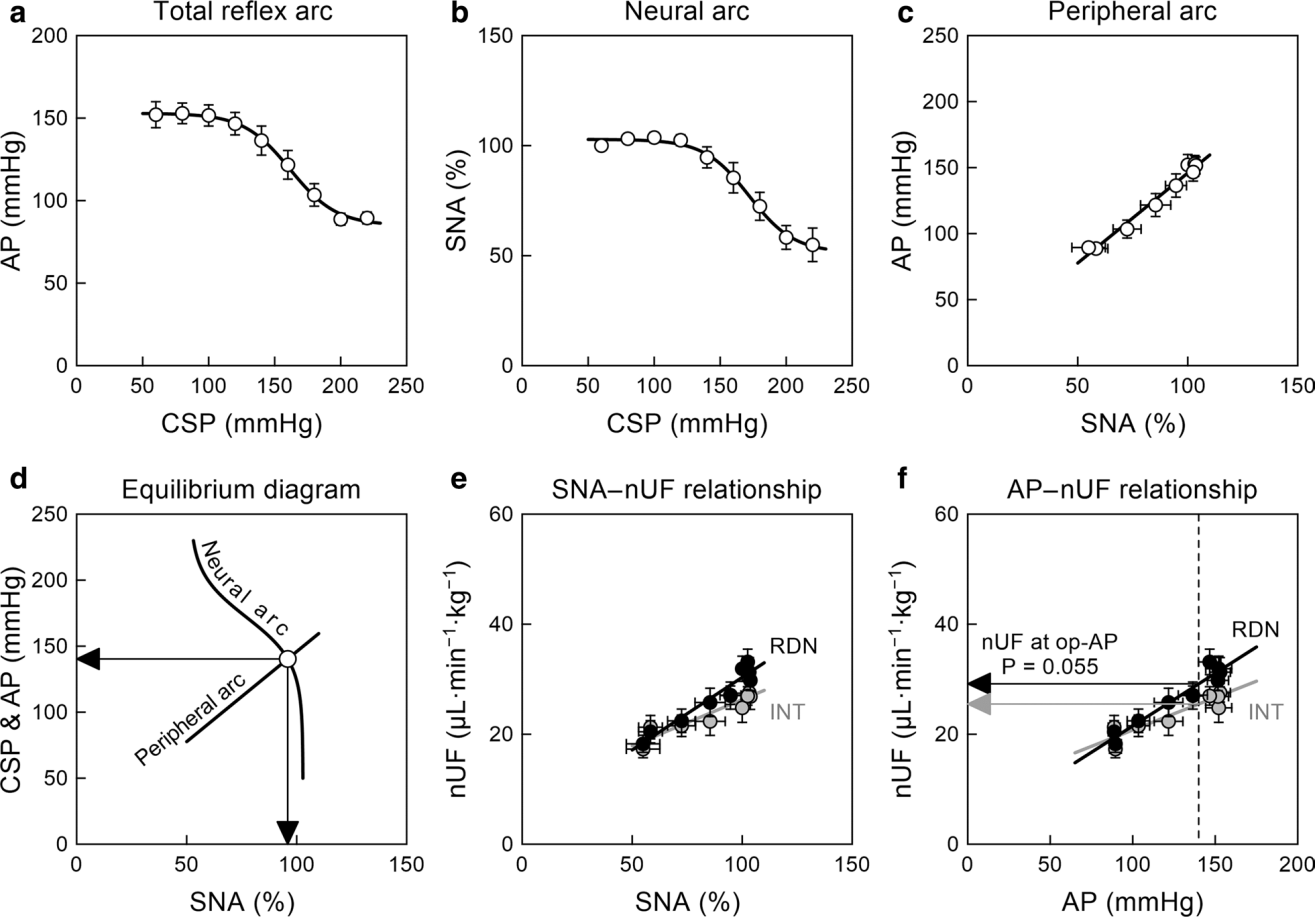
**a–c** Group-averaged static characteristics of the total reflex arc, neural arc, and peripheral arc, respectively, of the carotid sinus baroreflex. CSP, carotid sinus pressure; AP, arterial pressure; SNA, sympathetic nerve activity. **d** The baroreflex equilibrium diagram constructed from the fitted neural and peripheral arcs. Downward and leftward arrowheads indicate the operating points of SNA and AP, respectively. **e** The relationship of normalized urine flow (nUF) versus SNA in the intact (INT) and renal denervation (RDN) sides. **f** The relationship of nUF versus AP in the INT and RDN sides. The vertical dashed line indicates the operating-point AP (op-AP). The horizontal arrowheads indicate nUF at op-AP. The P value was determined by the Wilcoxon signed-rank test. Data are expressed as mean ± SE values (*n* = 9 rats)

Figure 2 and Table 1 summarize the open-loop static characteristics of the carotid sinus baroreflex. The total reflex arc (Fig. 2a) and the neural arc (Fig. 2b) approximated inverse sigmoid curves. The maximum SNA occurred at CSP steps in the range of 80–120 mmHg, and slightly exceeded 100%, because SNA at the CSP of 60 mmHg was defined to be 100%. The peripheral arc (Fig. 2c) approximated a straight line. In the baroreflex equilibrium diagram (Fig. 2d), the intersection between the neural and peripheral arcs provided the operating point.

**Table 1:** Parameters of the carotid sinus baroreflex open-loop characteristics

Total reflex arc	
* P*_1_, response range, mmHg	67.0 ± 7.7
* P*_2_, slope coefficient, mmHg^−1^	0.117 ± 0.026
* P*_3_, midpoint pressure, mmHg	155.2 ± 6.3
* P*_4_, minimum pressure, mmHg	87.0 ± 3.6
Neural arc	
* P*_1_, response range, %	52.9 ± 8.1
* P*_2_, slope coefficient, mmHg^−1^	0.102 ± 0.020
* P*_3_, midpoint pressure, mmHg	165.7 ± 5.8
* P*_4_, minimum value, %	51.2 ± 7.9
Peripheral arc	
Intercept, mmHg	1.6 ± 24.1
Slope, mmHg/%	1.448 ± 0.233
Operating-point parameters	
Operating-point SNA, %	94.9 ± 3.8
Operating-point AP, mmHg	138.3 ± 5.1

Data are expressed as mean ± SE (*n* = 9 rats)

*SNA* sympathetic nerve activity, *AP* arterial pressure

The SNA and nUF showed a positive correlation on both sides (Fig. 2e), and the regression slope tended to be greater on the denervated side (Table 2). The AP and nUF also showed a positive correlation on both sides (Fig. 2f). The regression slope of the AP–nUF relationship did not differ significantly between the intact and denervated sides. The vertical dotted line in Fig. 2f denotes the operating point of the AP estimated from the equilibrium diagram. The nUF at the operating-point AP, depicted in the leftward arrowhead, tended to be higher on the denervated than the intact side (Table 2). The percent increase of the nUF at the operating-point AP induced by RDN was 17.2 ± 7.2%.

**Table 2:** Effects of unilateral renal denervation (RDN) on normalized urine flow (nUF)

	INT	RDN	*P* value
Slope of nUF versus SNA, μL min^−1^ kg^−1^ %^−1^	0.168 ± 0.042	0.264 ± 0.059	0.055
Intercept of nUF versus SNA, μL min^−1^ kg^−1^	9.2 ± 3.8	3.8 ± 5.3	0.301
Slope of nUF versus AP, μL min^−1^ kg^−1^ mmHg^−1^	0.120 ± 0.031	0.179 ± 0.025	0.098
Intercept of nUF versus AP, μL min^−1^ kg^−1^	9.4 ± 3.6	4.4 ± 3.0	0.301
nUF at the operating-point AP, μL min^−1^ kg^−1^	25.3 ± 1.9	29.0 ± 1.8	0.055

Data are expressed as mean ± SE (*n* = 9 rats). INT, intact side; SNA, sympathetic nerve activity; AP, arterial pressure. P values were calculated by the Wilcoxon signed-rank test

Sodium and creatinine concentrations in the plasma and the cumulated urine were measured in eight out of nine rats (Table 3). Creatinine clearance, calculated using the nUF at the operating-point AP, did not differ significantly between the intact and denervated sides (2508 ± 277 vs. 2769 ± 485 μL min^−1^ kg^−1^, *P* = 0.547, *n* = 8). Fractional sodium excretion did not differ significantly between the intact and denervated sides (0.54 ± 0.16% vs. 0.73 ± 0.24%, *P* = 0.195, *n* = 8).

**Table 3:** Sodium and creatinine concentrations in plasma and urine

	Plasma	INT Urine	RDN Urine	*P* value
Sodium, mEq/L	140.9 ± 1.0	55.8 ± 8.0	61.5 ± 9.0	0.382
Creatinine, mg/dL	0.30 ± 0.01	29.3 ± 3.9	26.8 ± 4.0	0.250

Data are expressed as mean ± SE (*n* = 8 rats). INT, intact side; RDN, renal denervation side. P values were calculated by the Wilcoxon signed-rank test between urine values from INT and RDN sides

## Discussion

### Sympathetic activation and urine output response

Neural control of the kidney through renal innervation is important for body fluid volume control. Renal sympathetic activation exerts an antidiuretic effect via the release of renin, renal vasoconstriction, and sodium and water reabsorption [1]. During a hypotensive event, such as that induced by hemorrhage, a decrease in renal perfusion pressure and a baroreflex-mediated sympathetic activation act synergistically to reduce urine output and promote fluid retention. By contrast, an increase in renal perfusion pressure due to an AP elevation following PASA can counteract the antidiuretic effect via renal sympathetic innervation. We have demonstrated that urine output from the innervated kidney increases with SNA during PASA in WKY [3]. If the antidiuretic effect via renal innervation in SHR is powerful enough compared with systemic sympathetic control of the AP, the urine output could be reduced with increasing SNA during PASA. However, nUF on the intact side positively correlated with SNA during PASA in SHR (Fig. 2e). Hence, the pressure diuresis resulting from an AP elevation following PASA outweighed the antidiuretic effect via renal efferent innervation not only in WKY but also in SHR. The slope values of the SNA–nUF relationship were not directly compared between SHR and WKY, because the normalization of SNA could complicate the interpretation of the slope.

Sympathetic activation in SHR compared with WKY is to be briefly discussed. The carotid sinus baroreflex function in SHR resets to a higher input pressure range [14], which is confirmed by the high midpoint pressure in the neural arc (Table 1). Although high-CSP inputs can usually reduce SNA to approximately 10% or less in WKY [3, 15], the lower plateau of the neural arc was above 50% in the present study that suggests an incomplete sympathetic suppression in SHR. Furthermore, reductions of SNA and AP in response to the CSP step changes were transient compared with more sustained reductions observed in WKY [3]. The sustained AP reduction is mediated via the central pathway of unmyelinated C-fiber baroreceptors [16]. There are significantly fewer C-fiber baroreceptor axons in SHR compared with WKY [17], and nonderivative dynamic characteristics of the C-fiber central pathway are compromised in SHR [18]. As a result, SNA and AP did not show sustained responses to the CSP step changes in SHR. Nevertheless, the lower plateau of the total reflex arc was 87 mmHg (Table 1). Hence, the baroreflex-mediated sympathetic suppression can reduce AP to a normal pressure range in SHR if the baroreceptors are activated at adequately high pressures. Ganglionic blockade reduces AP in SHR to the same level as that in WKY [15], which further suggests that hypertension in SHR depends on high-resting SNA.

The slope of the AP–nUF relationship on the intact side was positive in SHR (Fig. 2f, Table 2), but the mean slope was less than 1/3 of that in WKY observed in our previous study (0.420 ± 0.081 μL min^−1^ kg^−1^ mmHg^−1^, *P* = 0.013 by the Mann–Whitney test) [3]. If the slope difference is mainly attributed to the ongoing neural control over the kidney, the slope on the denervated side may be comparable between SHR and WKY. However, the slope of the AP–nUF relationship on the denervated side in SHR (Table 2) remained less than 1/3 of that in WKY (0.552 ± 0.112 μL min^−1^ kg^−1^ mmHg^−1^, *P* = 0.001 by the Mann–Whitney test) [3]. Hence, the lower slope of the AP–nUF relationship in SHR compared with WKY is unlikely caused by the ongoing high renal SNA. The results are consistent with an earlier study that compared the effect of renal perfusion pressure on the UF in a denervated kidney between SHR and WKY [19]. The percent increase in nUF at the operating-point AP induced by RDN was not significantly different from that observed in WKY in our previous study (14.7 ± 6.0%, *P* = 0.780 by the Mann–Whitney test) [3].

The depression of renal function in SHR was not detected from the creatinine clearance and fractional sodium excretion, because these values were similar to those observed in WKY [3]. The results are in line with a previous study that indicated lack of significant differences in glomerular filtration rate or fractional sodium excretion between SHR and WKY at 14 weeks of age [20].

### RDN and hypertension

Although RDN has been explored as a device therapy for resistant hypertension, a blinded, randomized, sham-controlled trial SYMPLICITY HTN-3 did not show a significant reduction of systolic blood pressure (SBP) in patients with resistant hypertension 6 months after RDN as compared with those who underwent a sham procedure [21]. Although the reason for the lack of significant effects of RDN might be multifactorial, incomplete RDN was suspected [22]. A newer trial, SPYRAL HTN-OFF MED Pivotal, which used an improved device to achieve thorough and consistent RDN, demonstrated a significant SBP reduction in a denervated group compared with a sham procedure group in the absence of antihypertensive medications [23]. However, the reduction of 24-h SBP at 3 months after RDN was between − 6.4 and − 2.9 mmHg (95% confidence interval). Given that the pathophysiology of hypertension is heterogeneous, RDN may not be a panacea for all hypertensive patients [24]. It is necessary to identify subgroups of patients who benefit maximally from RDN based on an understanding of the effect of RDN on the AP regulation.

Since we did not prepare a proper control group of sham-operated SHR, whether unilateral RDN reduced AP was inconclusive in the present study. Five-min averaged AP and HR values measured under anesthesia but before the baroreceptor isolation procedure were 178.7 ± 8.7 mmHg and 417.3 ± 15.3 beats/min, respectively (*n* = 9). As a reference, the 5-min averaged AP and HR values measured under similar settings were 195.7 ± 4.9 mmHg and 421.8 ± 11.3 beats/min, respectively, in SHR without any prior operation (*n* = 12, randomly selected from our past studies). While the mean AP in SHR with unilateral RDN was numerically lower than that in SHR without any prior operation, the difference was not statistically significant by the Mann–Whitney test (*P* = 0.177). The difference in HR was not statistically significant, either (*P* = 0.722).

Although unilateral RDN has the merit that we can compare the urine output function between the intact and denervated kidneys under the same perfusion pressure and circulating humoral factors, it cannot provide information on the effect of bilateral RDN. Gao et al. [25] demonstrated that bilateral radiofrequency renal artery denervation in SHR with established hypertension produced a sustained reduction of SBP (171 ± 6 mmHg) compared with sham control (183 ± 4 mmHg) at 8 weeks after RDN. While the effect of renal artery denervation was significant, AP remained higher than the normal pressure range. An earlier study indicated that bilateral RDN in SHR delayed the development of hypertension by approximately 2 weeks, but did not affect the final AP level compared with sham-operated SHR [26]. In another study, bilateral RDN performed at 13 weeks of age reduced SBP of SHR [27]. However, SBP of SHR with bilateral RDN increased gradually beginning 21 weeks after RDN and reached a level equivalent to SBP of sham-operated SHR at 35 weeks after RDN. At this time point (48 weeks of age), plasma renin activity (PRA) of sham-operated SHR was higher than that of WKY. By contrast, PRA of SHR with bilateral RDN was reduced to a level comparable to WKY [27]. Hence, the reduction of PRA by bilateral RDN did not prevent the eventual development of hypertension in SHR.

A kidney cross-transplantation study indicated that normotensive recipients of SHR kidneys developed hypertension, and that the transplantation of normotensive kidneys reduced the blood pressure of hypertensive recipients [28]. These results suggest that kidneys have a fundamental role in the development of hypertension in SHR. One of the potential mechanisms for the impaired diuresis in SHR kidneys is a defect in dopamine receptor signaling [29]. Dopamine produced by renal proximal tubules serves as an intrarenal natriuretic hormone by decreasing tubular sodium transport. SHR kidneys show uncoupling between the D_1_-like receptor and its effector enzyme complex in proximal tubules, leading to a failure of dopamine to inhibit proximal tubular luminal sodium/hydrogen exchanger and sodium/potassium ATPase activity. Chronic inhibition of dopamine biosynthesis accelerates the development of hypertension in SHR [30].

It remains difficult to reconcile a) the fundamental role of the kidneys in developing hypertension in SHR and b) the fact that high-resting SNA may be required for maintaining hypertension in SHR. One possible explanation is that renal afferent signaling from the damaged kidney to the brain evokes systemic sympathetic activation [22]. Electrical stimulation of the renal afferent nerve activates neurons in the paraventricular nucleus projecting to the rostral ventrolateral medulla that serves as a center of sympathetic outflow [31]. However, the renal afferent mechanism alone may not be able to explain why SHR kidneys transplanted into normotensive recipients develop hypertension despite the total denervation. It has been shown that an increase in the sodium concentration in cerebrospinal fluid precedes an increase in AP induced by high-salt diet in SHR and Dahl salt-sensitive rats [32]. The plasma sodium concentration was not significantly different between SHR and WKY or between regular- and high-salt diets in that study. Given that pressure-dependent renal sodium excretion was depressed in parallel with renal urine output function in SHR compared with WKY [19], the difference in sodium handling—even though it is not reflected in the plasma sodium concentration—may cause central effects that increase SNA in SHR. A caveat to this interpretation is that the sodium concentration in cerebrospinal fluid is not different between WKY and SHR during regular-salt diet [32].

## Conclusions

The present study demonstrated that PASA increased urine output in SHR. Although sympathetic activation is generally considered to promote body fluid volume retention, sympathetic activation, when it accompanies an AP elevation, may promote urine output against the depressed renal urine output function in SHR. The percent increase in the nUF at the operating-point AP induced by the RDN was not different from that observed previously in WKY. Although high-resting SNA is required to maintain hypertension in SHR, its effect on the urine output function was not more potent than that in WKY.

## Data Availability

The datasets used and/or analyzed during the current study are available from the corresponding author on reasonable request.

## Abbreviations

AP: Arterial pressure
CSP: Carotid sinus pressure
HR: Heart rate
nUF: Normalized urine flow
PASA: Primary acute sympathetic activation
RDN: Renal denervation
PRA: Plasma renin activity
SBP: Systolic blood pressure
SHR: Spontaneously hypertensive rats
SNA: Sympathetic nerve activity
UF: Urine flow
WKY: Wistar–Kyoto rats
